# Job satisfaction and its related factors among emergency department physicians in China

**DOI:** 10.3389/fpubh.2022.925686

**Published:** 2022-07-22

**Authors:** Kang Li, Hongmei Chen, Zhen Tan, Xiaoxv Yin, Yanhong Gong, Nan Jiang, Fengjie Yang

**Affiliations:** ^1^Tongji Hospital, Tongji Medical College, Huazhong University of Science and Technology, Wuhan, China; ^2^School of Nursing, Wuchang University of Technology, Wuhan, China; ^3^Shenzhen University General Hospital, Shenzhen University, Shenzhen, China; ^4^Department of Social Medicine and Health Management, School of Public Health, Tongji Medical College, Huazhong University of Science and Technology, Wuhan, China; ^5^Department of Pediatrics, Tongji Hospital, Tongji Medical College, Huazhong University of Science and Technology, Wuhan, China

**Keywords:** job satisfaction, physicians, emergency department, China, cross-sectional study

## Abstract

**Background:**

Job satisfaction is recognized as an important factor affecting the performance and quality of medical services of emergency department physicians. However, little is known about the status of job satisfaction among emergency department physicians in China. This study aimed to explore the current level of job satisfaction and its associated factors among emergency department physicians in China.

**Methods:**

A nationwide cross-sectional survey was conducted in China from July to August 2018. A total of 10,457 emergency department physicians completed the questionnaire. The structured online questionnaire collected information on socio-demographic characteristics, work-related factors, work-family conflict, and job satisfaction. Student's *t*-test or ANOVA were used to compare the job satisfaction scores in different characters. The generalized linear model was used to investigate the related factors of job satisfaction among emergency department physicians.

**Results:**

The respondents' job satisfaction average score was 12.2 ± 3.6, of which 42.01% were satisfied of which the job. The results showed that emergency department physicians over 41 years old, with a higher income and working in central and western regions were positively associated with job satisfaction. In contrast, bachelor degree and above, fixed posts, long years of service, a high frequency of night shift, perceived shortage of physicians, perceived medical errors, and higher work-family conflict scores were negatively correlated with job satisfaction among emergency department physicians.

**Conclusion:**

Job satisfaction of emergency department physicians in China is low. It is suggested that hospital administrators could improve the job satisfaction of emergency department physicians by establishing an acceptable shift system, ensuring adequate emergency department staffing, increasing their income appropriately and alleviating work-family conflict.

## Introduction

Job satisfaction is a pleasurable or positive emotional state resulting from the appraisal of one's job or job experiences (1). It has been reported that the job satisfaction of emergency department physicians has a significant impact on job performance. High level of job satisfaction can improve employee productivity and creativity (2) and are also associated with an efficient organization (3). Besides, high level of job satisfaction contributes to the stability of the emergency medical team due to its significant correlation with a low turnover rate (4). Correspondingly, dissatisfaction with the job can lead to a lower sense of belonging to the organization and a higher willingness to resign (5), affecting the quality of medical services provided by emergency department physicians. In severe cases, it can also threaten the safety of patients' lives (6). Therefore, hospital administrators should explore the status of job satisfaction of emergency department physicians (7).

Although some studies have reported on the level of job satisfaction among emergency department staff, few have been conducted on emergency department physicians, especially in China. A survey conducted in Beijing revealed that 6.8% of emergency department physicians were satisfied or very satisfied with their job (8), much lower than in the developed countries (9–11). What's more, with the growing demand for emergency medical services and the worsening shortage of emergency department physicians, the job satisfaction of emergency department physicians in China will become worse (12). It has been reported that from 2005 to 2017, the number of emergency department attendances has tripled from 51.9 to 166.5 million (13). In large cities in China, the workforce shortage in the emergency department is severe, with annual staff turnover rates approaching 50% (14).

After years of research, numerous variables regarding the individuals and work-related factors are identified to be associated with job satisfaction. A survey conducted in the United States showed that age and sex were predictors of emergency department physicians' job satisfaction (15). Increased age and clinical hours worked per year were associated with low job satisfaction of emergency department physicians based on an investigation in Canada (10). In addition, other factors including income, marital status and frequency of night shift had significant effects on job satisfaction among emergency department physicians in Jordan (16). The conflict between work and family often puts emergency department physicians under high levels of pressure, which is also disadvantage to their job satisfaction (17, 18). Few studies reported the related factors of job satisfaction among emergency department physicians in China (8, 19), and they have been conducted in certain hospitals in a few provinces and cities. The representative data on a national scale is lacking. This study was conducted nationwide to gain a comprehensive understanding of job satisfaction among emergency department physicians in China. The aim of this study is to evaluate the current status of job satisfaction among emergency department physicians and explore its associated factors.

## Methods

### Ethics statement

Study approval was obtained from the Research Ethics Committee of Hainan Medical University (Approval Number: HYLL-2018-035). All emergency department physicians participating in the survey obtained informed consent, and all the data would be kept confidential.

### Study design

This national cross-sectional study was conducted from July to August 2018 with the assistance of the Medical Administration Bureau of the National Health Commission of the People's Republic of China.

### Participants and data collection

The Questionnaire Star (https://www.wjx.cn/), an online investigative tool in China, was used to collect data. The electronic questionnaire was posted on the emergency department physicians' working platform, inviting emergency department physicians to take part in the survey anonymously. Every seven days, the questionnaire link was re-posted to the working platform to remind emergency department physicians to respond until the survey was completed. Before participating in the survey, all respondents needed to read and agree to the electronic informed consent statement. Respondents could not submit the questionnaire unless it was completed. All data were saved and managed by the Questionnaire Star. A total of 15,288 emergency department physicians clicked on the link of the questionnaire during the survey and 10,457 completed the questionnaire. The completion rate was 68.40%.

### Measures

The electronic questionnaire was designed based on literature review and pre-surveyed among 30 emergency department physicians. Based on the pre-survey results, the questionnaire was modified to ensure its understandable. Next, it was applied to data collection in formal surveys.

The questionnaire covered socio-demographic characteristics, work-related factors, work-family conflict, and job satisfaction. Specifically, the socio-demographic characteristics included age, gender, educational level, marital status and geographical region. According to the China Health and Family Planning Statistical Yearbook, we divided China into three regions, the eastern region (11 provinces), the central region (8 provinces), and the western region (12 provinces) (20). Work-related factors covered authorized strength, way of appointment, monthly income, years of service, frequency of night shift, perceived shortage of physicians, and perceived medical errors. The question: “Do you think the current number of physicians in the emergency department can meet the needs of daily work?” was used to assess the perceived shortage of physicians. The perceived medical errors were examined by one question: “In the past 3 months, have you worried that you have made a serious medical error?”

The Work-Family Conflict Scale designed by Netemeyer et al. (21) was used to assess the work-family conflict of emergency department physicians. Five items made up the scale and a 5-point Likert scale ranging from 1 (strongly disagree) to 5 (strongly agree) was used to score each item. The total score ranged from 5 to 30. The higher the score, the more prominent the conflict between family and work. The scale has been proved to be reliable and valid in previous studies in China (22, 23). The Cronbach's α for the scale was 0.94 in this study.

The Leiden Quality of Work Questionnaire was used to assess the job satisfaction of emergency department physicians (24, 25). The scale consists of 6 items, including “If I had to choose, I'd choose this job again”/“I would like to change my job”/“I'm satisfied with my job”/“I would recommend this job to a friend”/“When I applied, this was the job that I wanted”/“I often have to do work that I'd rather not do”. Each item was scored on a 4-point Likert scale, ranging from 1 (totally disagree) to 4 (totally agree). The total score ranged from 4 to 24. A higher score reflected a higher level of job satisfaction and a score above the average was defined as being satisfied with the job (26). In this study, the scale had a Cronbach's alpha value of 0.86.

### Data analysis

The Statistical Analysis System (SAS) version 9.4 for Windows (SAS Institute Inc., Cary, NC, USA) was used to perform the statistical analyses. In descriptive statistics, we used frequencies and percentages to describe categorical variables. Means and standard deviations were used to describe continuous variables. Student's *t*-test or ANOVA were used to compare the job satisfaction scores of physicians in different characters, and the Satterthwaite test was conducted when the homogeneity test of variances was significant. Kolmogorov-Smirnov test was used to test the normality of job satisfaction. A generalized linear model was used to analyze the associated factors of job satisfaction of physicians in the emergency department because the distribution of job satisfaction was abnormal (K-S = 0.098611, *P* < 0.0100). All comparisons were two-tailed, and a *P* < 0.05 was determined to be statistically significant.

## Results

### Characteristics of the participants

The general characteristics of the participants are shown in Table 1. A total of 10,457 emergency department physicians participated in the survey, with an average age of 36.5 ± 7.5 years. The largest proportion of respondents was in the 31–41 years age group, making up 42.71%. 72.98% of the respondents were male and 27.02% were female. Nearly 75% of the respondents had a bachelor's degree. The marital status of the emergency department physicians was mainly married, accounting for 84.42%. Among them, physicians from the eastern region and the western region each accounted for about 36%. Approximately two-thirds of respondents had an establishment, and nearly three-quarters had a fixed position. Only 29.00% of emergency department physicians had a monthly income of more than 6,000 RMB and more than half worked night shifts 6–10 times a month. The years of service of the respondents were mainly more than 6 years (48.24%) and most of them (73.32%) perceived a shortage of emergency department physicians. Besides, 43.63% of the respondents perceived medical errors.

**Table 1:** Univariate analysis of job satisfaction of emergency department physicians (*n* = 10,457).

**Variables**	**Number** **(%)**	**Job satisfaction** **scores** **(mean** ±**SD)**	**Statistical values** **(*****F*****/*****t*****)**
**Socio-demographic variables**			
**Age**			45.38^**^
≤31	3,111 (29.75)	12.39 ± 3.58	
31–41	4,466 (42.71)	11.79 ± 3.55	
≥41	2,880 (27.54)	12.52 ± 3.56	
**Gender**			−8.39^**^
Male	7,632 (72.98)	11.99 ± 3.62	
Female	2,825 (27.02)	12.64 ± 3.42	
**Educational level**			99.62^**^
Associate degree or below	1,684 (16.10)	13.28 ± 3.62	
Bachelor degree	7,789 (74.49)	11.94 ± 3.51	
Master degree or above	984 (9.41)	12.11 ± 3.64	
**Marital status**			4.06^**^
Unmarried/other	1,629 (15.58)	12.51 ± 3.71	
Married	8,828 (84.42)	12.11 ± 3.55	
**Geographical region**			
Eastern region	3,807 (36.41)	11.96 ± 3.60	24.70^**^
Central region	2,830 (27.06)	12.56 ± 3.56	
Western region	3,820 (36.53)	12.09 ± 3.54	
**Work-related variables**			
**Authorized strength**			−3.09^*^
Yes	6,714 (64.21)	12.09 ± 3.56	
No	3,743 (35.79)	12.31 ± 3.60	
**Way of appointment**			7.97^**^
Rotate	2,511 (24.01)	12.66 ± 3.63	
Fixed post	7,946 (75.99)	12.01 ± 3.55	
**Monthly income (RMB)**			0.84
≤4,000	3,862 (36.93)	12.23 ± 3.67	
4,001–6,000	3,562 (34.06)	12.13 ± 3.51	
≥6,001	3,033 (29.00)	12.14 ± 3.53	
**Years of service**			63.01^**^
≤1	1,448 (13.85)	12.98 ± 3.51	
1–5	3,965 (37.92)	12.29 ± 3.57	
≥6	5,044 (48.24)	11.83 ± 3.56	
**Frequency of night shift (per month)**			189.76^**^
0–5	2,033 (19.44)	13.45 ± 3.59	
6–10	5,633 (53.87)	12.04 ± 3.47	
≥11	2,791 (26.69)	11.50 ± 3.54	
**Perceived shortage of physicians**			35.48^**^
No	2,790 (26.68)	14.11 ± 3.39	
Yes	7,667 (73.32)	11.46 ± 3.38	
**Perceived medical errors**			−24.85^**^
Yes	4,562 (43.63)	11.22 ± 3.33	
No	5,895 (56.37)	12.90 ± 3.59	

** *p < 0.001 level (two-tailed)*.

* *p < 0.05 level (two-tailed)*.

### Job satisfaction of emergency department physicians

The job satisfaction of emergency department physicians with different characteristics is presented in the Table 1. The average score of the job satisfaction of respondents was 12.2 (SD = 3.6). 42.01% of the respondents were satisfied with the job. In addition, the univariable analysis shows that there were significant differences in the job satisfaction among emergency department physicians in terms of age, gender, educational level, marital status, geographical region, authorized strength, way of appointment, years of service, frequency of night shift, perceived shortage of physicians and perceived medical errors.

Factors associated with the job satisfaction among emergency department physicians are presented in Table 2. The emergency department physicians over 41 years old showed higher job satisfaction (*P* = 0.0006). In comparison to emergency department physicians with vocational diploma or below, physicians attending a bachelor degree (*P* < 0.0001) and master degree or above (*P* < 0.0001) were less satisfied with their job. Besides, physicians in the eastern region had lower job satisfaction than those in the central region (*P* < 0.0001) and western region (*P* < 0.0001). In terms of work-related variables, physicians with a fixed post (*P* = 0.0002), a work experience of 1–5 years (*P* = 0.0331) and more than 6 years (*P* < 0.0001), 6–10 night shifts (*P* < 0.0001) and ≥11 night shifts (*P* < 0.0001) per month presented lower job satisfaction scores. Emergency department physicians with a monthly income of between 4,001 and 6,000 RMB (*P* = 0.0122) and more than 6,000 RMB (*P* = 0.0315) had higher job satisfaction than those with ≤4,000 RMB. Besides, physicians who perceived the shortage of physicians (*P* < 0.0001) and medical errors (*P* < 0.0001) were less satisfied with their job. Work-family conflict (*P* < 0.0001) also had negative impacts on job satisfaction.

**Table 2:** Generalized linear regression examining factors associated with job satisfaction.

**Variables**	β	**SE**	***t***	***p*** **-value**
**Socio-demographic variables**				
**Age (ref: ≤31)**				
31–41	0.057	0.078	0.74	0.4613
≥41	0.323	0.095	3.42	0.0006
**Gender (ref = male)**				
Female	0.085	0.066	1.30	0.1927
**Educational level (ref = vocational diploma or below)**				
Bachelor degree	−0.657	0.082	−8.01	<0.0001
Master degree or above	−0.823	0.124	−6.63	<0.0001
**Marital status (ref = unmarried/other)**				
Married	0.091	0.085	1.07	0.2854
**Geographical region (ref = Eastern region)**				
Central region	0.399	0.074	5.41	<0.0001
Western region	0.268	0.067	4.01	<0.0001
**Work-related variables**				
**Authorized strength (ref = yes)**				
No	−0.007	0.064	−0.11	0.9115
**Way of appointment (ref = rotate)**				
Fixed post	−0.266	0.072	−3.67	0.0002
**Monthly income (RMB) (ref: ≤4,000)**				
4,001–6,000	0.174	0.069	2.51	0.0122
≥6,001	0.164	0.076	2.15	0.0315
**Years of service (ref: ≤1)**				
1–5	−0.201	0.094	−2.13	0.0331
≥6	−0.500	0.104	−4.83	<0.0001
**Frequency of night shift (per month) (ref: 0–5)**				
6–10	−0.611	0.077	−7.94	<0.0001
≥11	−0.700	0.089	−7.91	<0.0001
**Perceived shortage of physicians (ref = no)**				
Yes	−1.249	0.069	−18.13	<0.0001
**Perceived medical errors (ref = no)**				
Yes	−0.795	0.059	−13.49	<0.0001
Work-family conflict	−0.398	0.008	−50.80	<0.0001

*F = 378.48, p < 0.0001. R^2^ was 0.395*.

*Ref is a reference*.

## Discussion

This study reported the current situation of job satisfaction among emergency department physicians in China and explored its relationship with both socio-demographic characteristics and work-related factors. According to our study, less than half of the emergency department physicians were satisfied with their jobs, much lower than in other countries, including the United States (65.2%) (9) and Canada (75.5%) (10). This finding suggested that more attention should be paid to improving emergency department physicians' job satisfaction in China.

In the present study, emergency department physicians aged more than 41 years old had higher job satisfaction. This result was consistent with the previous study (27) and highlighted the importance of enhancing job satisfaction of young emergency department physicians. In addition, emergency department physicians with bachelor degrees or above were less satisfied with the job than those with vocational diplomas or below. It may be because emergency department physicians with higher education levels have more expectations of their careers. They are less likely to reach a state of satisfaction with their work (28). The higher the educational level, the greater the likelihood that the emergency department physicians will feel dissatisfied with daily routine tasks (29, 30). Therefore, hospital administrators should provide more opportunities for highly educated emergency department physicians to fulfill their job expectations.

Furthermore, emergency department physicians working in the eastern region showed lower job satisfaction than physicians in the central and western regions. This may be due to the heavier workload of emergency physicians in the eastern region. It is estimated that in 2018, the average number of patient visits undertaken per day by each physician in the eastern region was 8.1, higher than 5.6 in the central region and 6.5 in the western region (31). According to the job demand-control model theory, workload, as one of the key determinants of demand, is considered a source of stress and dissatisfaction (32). It has been reported that high levels of physician workload are significantly associated with reduced job satisfaction (33, 34). As a result, hospital administrators and policymakers should reduce the workload of emergency department physicians in the eastern region.

Our study also observed that work-related factors were significantly associated with the job satisfaction of emergency department physicians. Firstly, emergency department physicians with fixed posts seemed to be less satisfied with the job. This may be related to the work content and working environment of emergency department physicians. Emergency department physicians not only handle patients with acute and critical illnesses, but also work in a chaotic environment that is prone to violence (35, 36). Physicians with fixed posts means they have to be faced with the poor working environment all the time, which contributes to low job satisfaction. Secondly, those with long years of service possessed lower job satisfaction in this study. According to the previous study, emergency department physicians with long years of service reported more working hours, which could result in emotional exhaustion and fatigue (37). It has been validated that emotional exhaustion and fatigue are negatively associated with job satisfaction. Thirdly, the high frequency of night shifts and a perceived shortage of physicians were detrimental factors for job satisfaction. Similar results were observed in other studies (38–40) and it can be explained by the increasing workload resulting from the above two factors. Moreover, the long-term heavy workload can also damage the physical health of emergency department physicians, which in turn reduces their job satisfaction. In order to improve the job satisfaction of emergency department physicians, hospital managers should improve their working environment, develop a scientific night shift system and ensure adequate staffing.

This study also found that physicians with a monthly income of between 4,001 and 6,000 RMB and more than 6,000 RMB had higher job satisfaction. The result is consistent with previous research (27, 28, 41). According to the effort-reward imbalance theory (42), income is an important component of emergency department physicians' perceived rewards for their work. The lower the income, the more likely emergency department physicians are to lack reciprocity between effort and reward, resulting in them being in a state of emotional distress and more likely to be dissatisfied with the job (43). In contrast, emergency department physicians with a higher income are easier to perceive a balance between efforts and rewards in work, resulting in higher job satisfaction (44, 45). Accordingly, increasing the income of emergency department physicians may help improve their job satisfaction.

In our study, perceived medical errors were negatively correlated with job satisfaction. Undoubtedly, medical errors could lead to patients' dissatisfaction with treatment, affecting the doctor-patient relationship (46). According to previous research (26, 47), a bad doctor-patient relationship was to the disadvantage of the job satisfaction of emergency department physicians. In addition, medical errors can also make physicians less confident about their future career development, resulting in dissatisfaction with the job. Consistent with prior research findings (48, 49), work-family conflict had a negative influence on job satisfaction. Role stress theory, which assumed a disagreement between the requirements and values of an individual's work role and family role could explain the effects of work-family conflict on job satisfaction (50). When a physician thought his or her energy and time participating in family activities were interfered with by work, he or she might feel unhappy and dissatisfied with the job. Consequently, a more reasonable work allocation and vacation system for physicians to better balance work and life is necessary.

The completion rate of this study was 68.40%, which was higher than that of similar web-based survey studies (51–55). However, nearly one-third of emergency department physicians did not complete the questionnaire, possibly due to the following three reasons. Firstly, emergency department physicians are very busy in China, typically working 40–50 h per week (56). At the same time, they also have to bear the burden of family (41), so some emergency department physicians did not have sufficient time to participate in the investigation. Secondly, participants were required to complete all of the questions before submitting. Some emergency department physicians failed to submit the questionnaires because they missed some questions. Thirdly, quality control questions were randomly set in the questionnaire, and data for incorrectly completed these questions were not included in the analysis.

## Strengths and limitations

To the best of our knowledge, this study was the first to investigate the job satisfaction of emergency department physicians on a nationwide scale. The related factors of job satisfaction will provide a valuable basis for formulating interventions. However, there were some limitations in the study. Firstly, this study was a cross-sectional study and clear causal conclusions could not be drawn. Further prospective research is needed. Secondly, nearly one-third of emergency department physicians in this study did not complete the questionnaire, which may cause some impacts on the results of the study. However, the incompletion rate is similar to previous studies of web-based survey of other population (51–55). In the future, we can apply incentives and other measures to further increase the completion rate of web-based surveys to obtain more accurate results. Thirdly, the results only reflected the situations in China. Due to different implementation of policy and health system reforms in different countries, conclusions might not adapt to other countries.

## Conclusion

The job satisfaction of emergency department physicians in China is at a low level. Based on the survey results, hospital administrators need to take targeted measures to improve the job satisfaction of emergency department physicians. On the one hand, reasonable work arrangements and adequate staffing of the emergency department are needed. On the other hand, an acceptable shift system and a scientific reward mechanism are also helpful to improve the job satisfaction of emergency department physicians.

## Data availability statement

The datasets generated during and analyzed during the current study are available from the corresponding author on reasonable request.

## Ethics statement

The study was approved by the Research Ethics Committee in Hainan Medical University, Hainan, China (Approval Number: HYLL-2018-035). All participants provided their informed consent before participating in this study.

## Author contributions

KL, XY, NJ, and FY participated in the design of the study. XY, ZT, and YG collected the data. KL, HC, ZT, and NJ were involved in the data analysis. XY, ZT, NJ, and FY were involved in the supervision of the study. KL, XY, and NJ participated in the manuscript writing. XY, NJ, and FY contributed to the critical revisions of the manuscript. All authors read and approved the final manuscript.

## Funding

The study was supported by the Major Science and Technology Projects under Grant Number ZDKJ202004 and Key Research and Development Program under Grant Number ZDYF2020112, Department of Science and Technology of Hainan Province; Natural Science Foundation of Shenzhen University General Hospital (No. SUGH2020QD015); Shenzhen Natural Science Fund (the Stable Support Plan Program, No. 20200826225552001). The funding resource had no role in study design; data collection, analysis and interpretation of data; writing of the report; or in the decision to submit the article for publication.

## Conflict of interest

The authors declare that the research was conducted in the absence of any commercial or financial relationships that could be construed as a potential conflict of interest.

## Publisher's note

All claims expressed in this article are solely those of the authors and do not necessarily represent those of their affiliated organizations, or those of the publisher, the editors and the reviewers. Any product that may be evaluated in this article, or claim that may be made by its manufacturer, is not guaranteed or endorsed by the publisher.
